# The predictive value of NLR, PLR and MLR in the differential diagnosis of benign uterine diseases and endometrial malignant tumors

**DOI:** 10.1007/s12672-024-00956-8

**Published:** 2024-03-31

**Authors:** Lin Qin

**Affiliations:** Senior Department of Obstetrics & Gynecology, The Seventh Medical Center of PLA General Hospital, Beijing, China

**Keywords:** Endometrial cancer, NLR, PLR, MLR

## Abstract

**Objective:**

To explore the application of neutrophils to lymphocytes ratio (NLR), platelet to lymphocyte ratio (PLR) and monocyte to lymphocyte ratio (MLR) in the differential diagnosis of benign uterine diseases and endometrial malignant tumors.

**Methods:**

80 patients with endometrial malignant tumor diagnosed in our hospital from January 2019 to December 2022 were selected as the study group, and 74 patients with benign uterine diseases confirmed by pathology in our hospital during the same period were randomly selected as the control group. The differences of NLR, PLR and MLR in the peripheral blood of patients in each group were compared, and the value of individual indicators in the diagnosis of endometrial malignant tumor was evaluated using the Receiver Operating Characteristic (ROC) curve.

**Results:**

In peripheral blood, the NLR, PLR and MLR value in patients with endometrial cancer (EC) were significantly higher than those in patients with benign uterine diseases (P < 0.05). The area under the curve (AUC) of NLR, PLR, MLR in peripheral blood were 0.777, 0. 675 and 0.698. The best cutoff values were 2.02, 132.06 and 0.166. The sensitivity and specificity were 62.5% and 79.7%, 62.5% and 63.5%, 81 3% and 47.3%. The combination of these three indicators can significantly improved the diagnostic efficiency in endometrial cancer (AUC = 0.780), and the sensitivity and specificity were 60% and 83.8%.

**Conclusions:**

In peripheral blood, NLR, PLR and MLR have certain diagnostic value in the differential diagnosis of endometrial cancer. When NLR, PLR and MLR are elevated, we should be alert to the occurrence of endometrial malignant tumors, and the combined diagnostic efficiency is high.

## Introduction

Endometrial cancer is a common gynecological malignant tumor [1, 2], ranking the second in the malignant tumors of female reproductive system in China, and the incidence rate has shown a younger trend in recent years. Ultrasound plays a certain role in the diagnosis of endometrial carcinoma, and endometrial histopathological examination is the gold standard for diagnosis. However, curettage surge is an operation, which causes great damage to the patient, and also needs to bear the risk of anesthesia. Therefore, it is particularly important to find a simple and reliable index for early diagnosis of endometrial cancer. Research shown that inflammation can promote tumor angiogenesis, tumor cell proliferation and inhibit tumor cell apoptosis, so tumor is closely related to inflammatory response, and some inflammatory response indicators are related to tumor prognosis [3, 4]. NLR, PLR and MLR, as the clinical indicators of inflammation in the body, are relatively intuitive, cheap and convenient, which can comprehensively reflect the immune status and indicate the prognosis of the patients [5, 6]. Because the histopathological examination is necessary for the confirmation of the diagnosis of endometrial cancer, the usefulness of NLR, PLR, and MLR for clinical practice is unclear. This article aims to explore the diagnostic value of NLR, PLR and MLR in benign uterine diseases and endometrial malignant tumors, and provide theoretical basis for clinical diagnosis, treatment and prognosis.

## Materials and methods

### Subjects

80 patients with endometrial malignancies diagnosed in our hospital from January 2019 to December 2022 were collected as the study group, aged 38–83 years, with an average age of 58.79 ± 9.99 years. Inclusion criteria: the patient underwent diagnostic curettage due to suspected endometrial lesions, and the results were all from our hospital. And the endometrial malignant lesions confirmed by postoperative pathology. Exclusion criteria: ① taking medication, radiotherapy or chemotherapy before operation; ② It was complicated with cardio-cerebrovascular disease, liver, kidney, spleen and other important organ diseases, and was in the stage of acute infection; ③ Immunosuppression patients: such as Acquired Immune Deficiency Syndrome (AIDS) patients, long-term glucocorticosteroid treatment, immunosuppression patients; ④ Patients with incomplete medical records and missing laboratory test results. There were 69 cases of endometrial adenocarcinoma (69/80,86.25%), 4 cases of serous adenocarcinoma of endometrium (4/80, 5%), 3 cases of endometrial adenosquamous carcinoma (3/80, 3.75%), and 4 cases of endometrial clear cell carcinoma (4/80, 5%) in this group. 74 patients with benign uterine diseases in our hospital during the same period were randomly selected as the control group, with an average age of 50.35 ± 11.76 years, aged 21–80 years. There were 27 cases of endometrial hyperplasia (27/74, 36.49%), 36 cases of endometrial polyps (36/74, 48.65%) and 11 cases of submucous myoma of uterus (11/74, 14.86%) in this group. The pathology diagnosis relied on diagnostic curettage. This study was approved by the Ethics Committee of our hospital, batch number: 2023KY017-KS001.

### Methods

The test results of all the subjects were the data of our hospital. The routine blood test was carried out by SYSMEX automatic blood and body fluid analyzer (no.XN9000, Ximeikang Medical Electronics Co., Ltd, Shanghai). The routine blood test was reported within 2 weeks before treatment. We checked the electronic case, recorded the clinical data of the subjects at the initial diagnosis, including onset age, the number of white blood cell (WBC), neutrophil (NEU) count, lymphocyte (LYM) count, monocyte (MON) count, platelet (PLT) count, then calculated NLR, PLR and MLR, and compared the test results of the two groups of patients.

### Statistical analysis

SPSS 23.0 software was used for statistical analysis. Compliance with normal distribution was verified with the Shapiro–Wilk test, and the differences were assessed using Student’s t-test. For the measurement data that do not conform to the normal distribution, the median and quartile were used, and the Mann–Whitney U test was used for inter-group comparison. The predictive value was evaluated using the Receiver Operating Characteristic (ROC) curve analysis. The difference was statistically significant (P < 0.05).

## Results

### Comparison of NLR, PLR and MLR between endometrial cancer group and benign uterine diseases group

The levels of NLR, PLR and MLR in endometrial cancer group (study group) were 2.16 (1.68, 3.49), 158.41 (115.00, 203.85) and 0.22 (0.17, 0.32) respectively, which were higher than 1.51 (1.00, 1.95), 118.75 (91.13, 165.38) and 0.17 (0.13, 0.22) in benign uterine diseases group (control group), with statistically significant differences (P < 0.05) (Table 1).

**Table 1:** Comparison of various indicators of benign uterine diseases and endometrial malignant tumors

Index	Study group (n = 80)	Control group (n = 74)	Z	P-value
NLR	2.16 (1.68, 3.49)	1.51 (1.00, 1.95)	− 5.931	< 0.001
PLR	158.41 (115.00,203.85)	118.75 (91.13,165.38)	− 4.245	< 0.001
MLR	0.22 (0.17,0.32)	0.17 (0.13,0.22)	− 3.754	< 0.001

### ROC curve of efficacy evaluation of NLR, PLR and MLR in diagnosis of endometrial cancer

**Fig. 1:**
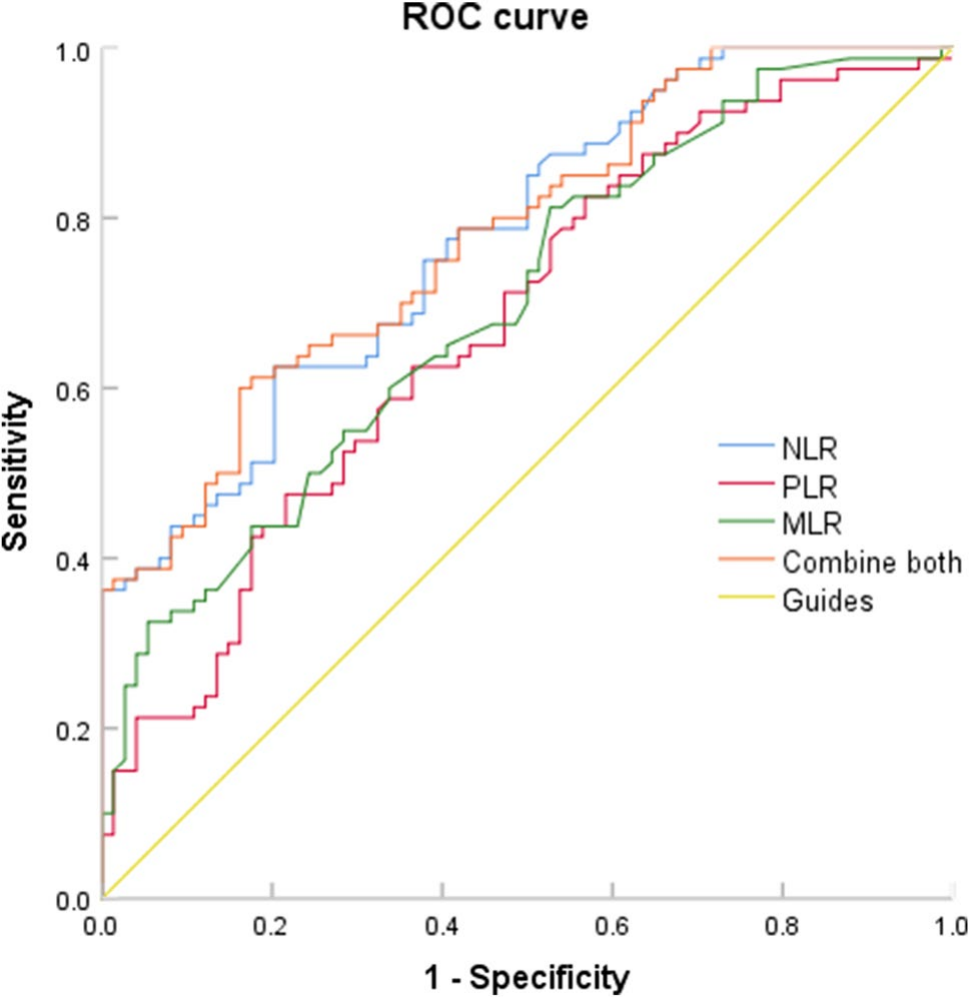
ROC curve of differential diagnosis of benign uterine diseases and endometrial malignant tumors by NLR, PLR, MLR and combined test

The analysis shown that NLR, PLR and MLR all showed high diagnostic performance in endometrial carcinoma, and NLR had the highest diagnostic efficiency. When the Cut-off value of NLR was set to 2.02 according to the Yoden index, the sensitivity was 62.5%, the specificity was 79.7%, and the area under the curve (AUC) was 0.777. When the Cut-off value of PLR was set to 132.06, the sensitivity was 62.5%, the specificity was 63.5%, and AUC was 0.675. When the Cut-off value of MLR was set to 0.166, the sensitivity was 81.3%, the specificity was 47.3%, and AUC was 0.698. However, the combination of these three indicators can significantly improved the diagnostic efficiency of EC (AUC = 0.780), and the difference was statistically significant (P < 0.05). The sensitivity and specificity were 60% and 83.8% respectively (Table 2 and Fig. 1).

**Table 2:** Differential diagnostic efficacy of peripheral blood NLR, PLR, MLR and combined test in benign uterine diseases and endometrial malignant tumors

Items	AUC	Cutoff	Sensitivity (%)	Specifity (%)	95%*CI*	P value
NLR	0.777	2.02	62.5	79.7	0.706–0.848	< 0.001
PLR	0.675	132.06	62.5	63.5	0.591–0.760	< 0.001
MLR	0.698	0.166	81.3	47.3	0.617–0.780	< 0.001
Combine test	0.780		60	83.8	0.710–0.851	< 0.001

## Discussion

In 1963, Virchow first linked cancer with chronic inflammation [7]. With the in-depth study of tumor mechanism, more and more studies have confirmed that inflammation is closely related to tumor. In the tumor microenvironment, tumor cells secrete various cytokines, chemokines and inflammatory cells, and inflammatory cells promote the occurrence, development and distant spread of tumor by promoting cell proliferation, inducing angiogenesis, and inhibiting cell apoptosis. In view of the close relationship between inflammation and tumor, tumor-related inflammatory markers have gradually become a research hotspot in recent years. NLR, PLR and MLR are better indicators of systemic inflammation [8, 9]. According to research reports, NLR, PLR and MLR played an important role in the diagnosis, prognosis and recurrence of tumors [10–13]. At the same time, routine blood test is an item for all patients to see a doctor and conduct routine examination before operation, without extra cost, so NLR, PLR and MLR are simple, effective, repeatable and promising predictors of diagnosis and treatment, disease judgment and prognosis of endometrial cancer patients. In recent years, many scholars have devoted themselves to studying the practical value of NLR in the diagnosis and treatment of various cancers, judging the prognosis of the disease and the effect of surgery or chemotherapy. This study analyzed the level changes of NLR, PLR and MLR in benign uterine diseases and endometrial malignant lesions, and discussed their clinical value in the diagnosis of endometrial cancer, in order to provide reference for the auxiliary diagnosis and treatment monitoring of NLR, PLR and MLR in endometrial cancer in the future.

NLR is a combination of neutrophils and lymphocyte counts, which is used as a representative index of systemic inflammation and is considered as a balance indicator of tumorigenic inflammatory response and anti-tumor inflammatory response. The increase of NLR in patients means the relative increase of neutrophils and the decrease of lymphocytes, thus promoting the development of inflammation towards tumorigenic inflammation. In addition to the secretion of vascular endothelial growth factor (VEGF) by neutrophils, the overexpression of VEGF can promote tumor angiogenesis and distant metastasis. In addition, the carcinogen secreted by neutrophils can stimulate the release of tumor tissue and make tumor tissue highly invasive. Increased neutrophils or decreased lymphocytes will inhibit the function of lymphokine-activated killer cells and enhance the tendency of distant metastasis. In addition, neutrophils are also involved in inducing tumor suppressor gene mutation, degrading immunoglobulin, degrading receptor and complement, and promoting the proliferation and differentiation of tumor cells. Lymphocytes are the smallest white blood cells. Lymphocytes are considered to play an important role in the natural immune defense of malignant tumors. The relative reduction of lymphocytes suggests that CD8 cytotoxic lymphocytes have a reduced function of killing tumor cells, the anti-tumor response mediated by T lymphocytes is weakened, and the anti-tumor ability of the body is reduced. The decrease of lymphocyte count in the blood has been determined as an independent prognostic factor for a variety of cancers [14]. Some studies had confirmed that the relative lymphocyte proliferation before treatment can improved the efficacy of radiotherapy and chemotherapy for malignant tumors [15]. Some studies had shown that among patients with recurrent ovarian cancer, high NLR was associated with poor prognosis [16, 17]. Prabawa et al. pointed out that NLR predicted the stage of cervical cancer, showing a significant positive correlation, with NLR cutoff value of 3.38, sensitivity of 82%, and specificity of 71% [18]. Catabay et al. research shown that [19] NLR was an independent prognostic indicator of malignant tumors. Ceran et al. shown that NLR > 3.83 was negatively correlated with progression-free survival (PFS) and overall survival (OS) in patients with ovarian cancer [20]. Abu-Shawer et al. shown that NLR had a certain role in evaluating the progression and metastasis of endometrial cancer [13]. Acmaz et al. shown than the boundary value of NLR was 2.89 in the endometrial cancer group [21]. Cummings et al. reported that the best cut-off value of NLR was 2.4, and the area under the curve was 0.616 [22]. However, Kurtoglu et al. shown that there was no statistical difference in NLR between patients with benign uterine diseases and endometrial cancer [23]. This study shown that NLR had diagnostic efficacy for endometrial cancer, with a critical value of 2.02, sensitivity of 62.5% and specificity of 79.7%. Haruma et al. shown that the best cutoff value of NLR for PFS was 2.41, sensitivity was 62.7%, specificity was 56.1%, and the best cutoff value of NLR for OS was 2.70, sensitivity was 69.7%, and specificity was 67.6% through the study on the prognosis of endometrial cancer [24]. Li et al. shown that the NLR truncation value was 4.68, and the OS difference in 5 years was significant [25]. Prognosis was not analyzed in this study, but can be evaluated in the later stage.

PLR is an important marker reflecting the degree of systemic inflammation, including two blood cell parameters of platelets and lymphocytes, and is an inflammatory marker reflecting the condition of tumor patients [26]. Janowska-Wieczorek et al. shown that platelets had the ability to deliver a variety of angiogenic factors to tumors and could stimulate tumor cells to express angiogenic factors [27]. In the diagnosis of ovarian cancer, PLR could cooperate with NLR, and when PLR > 231, it was negatively correlated with PFS and OS [20]. A systematic review and meta-analysis included 9 articles comprising 3390 patients, which resulted that elevated NLR and PLR during pretreatment were biomarkers of poor prognosis in patients with EC [28]. This study shown that PLR had diagnostic efficacy for EC, with a critical value of 132.06, sensitivity of 62.5%, and specificity of 63.5%. When PLR is higher than this threshold before treatment, it is necessary to be alert to endometrial cancer.

Monocyte is a subtype of WBC, the largest cell in the blood, which play an important part in the defense mechanism. Monocytes have the function of synthesizing inflammatory factors, and lymphocytes are the cells that regulate immunity. The ratio of MLR can reflect the inflammatory state of the body. Peripheral blood mononuclear cells are closely related to Tumor-Associated Macrophages (TAMs), play an immunosuppressive role in tumor cells, promote tumor cell infiltration, growth and angiogenesis, and create a good microenvironment for tumor occurrence [29, 30]. Monocyte elevation is associated with poor prognosis of various tumors [31, 32]. As a new inflammatory predictor, MLR had been found to be even better than NLR and PLR in the evaluation of inflammatory status of some diseases [33]. Some studies shown that PLR, NLR and MLR in patients with liver cancer, colorectal cancer and other malignant tumors were increased, and their level changes could be used to evaluate the prognosis [4, 34]. Eo et al. shown that MLR was an independent factor to predict the prognosis and survival rate of endometrial carcinoma [35]. Song et al. shown that high MLR were independent prognostic factors for disease-free survival and overall survival in patients with non endometrioid endometrial cancer, and the optimal cut-off value of MLR was 0.191 [36]. Njoku et al. Shown that absolute lymphocyte count, NLR, and MLR were associated with adverse clinico-pathologic factors, but not overall, cancer-specific or recurrence-free survival in the multivariable analysis from endometrial cancer [37]. Cong et al. shown that NLR, PLR, and MLR were independent prognostic markers for OS in EC patients, and the combined predictive value was higher than any single prediction among the three. The critical value of NLR was 2.14, PLR was 131.82, and MLR was 0.22 [38]. Holub et al. shown that NLR, MLR and lymphopenia proved to be independent unfavorable prognostic factors in EC patients staged I-III FIGO, and the critical value of NLR was 2.2, MLR was 0.18 [39]. This study shown that MLR had diagnostic efficacy for endometrial cancer, with a critical value of 0.166, sensitivity of 81.3%, and specificity of 47.3%.

This study divided patients into two groups based on pathological diagnosis: benign uterine diseases and endometrial malignant tumors. The NLR, PLR, and MLR of each group were detected, and the results showed that NLR, PLR, and MLR were higher in endometrial malignant tumors than in benign uterine diseases, which had statistical significance. On the basis of this study, the ROC curves of these three indicators were further drawn to evaluate their diagnostic value for endometrial malignant tumors. The optimal cutoff value was taken as the critical point, which was 2.02, 132.06, and 0.166, respectively. The AUC of the combined detection was 0.78, which was higher than that of the single indicator, indicating an improved prediction accuracy, with specificity of up to 83.8%. From this, it can be concluded that the combined detection of NLR, PLR, and MLR can more effectively distinguish between benign and malignant endometrial lesions, help improve the accuracy of endometrial malignant tumors diagnosis, and compensate for the shortcomings of the trauma caused by diagnostic curettage and easy missed diagnosis.

This study is a retrospective analysis of data. The included research data are obtained from clinical cases. There may be incomplete inclusion or inaccurate and biased data collection. This study is a single-center study with a small sample size, which may cause some errors and deviations in the results. The survival and prognosis of endometrial carcinoma were not followed up in this study. Therefore, more and more prospective studies are needed to confirm it in the future.

## Conclusions

To sum up, the NLR, PLR and MLR of endometrial cancer are higher than those of benign uterine diseases, which have certain diagnostic value and are expected to predict endometrial cancer. In addition, NLR, MLR and PLR are easily obtained hematological markers before treatment, which have the advantages of simplicity, convenience and low cost, and do not increase the physical pain and economic burden of patients. The combination of NLR, MLR and PLR has high sensitivity and specificity in the diagnosis of endometrial cancer, which can provide important reference, so as to better provide reference basis for clinical research.

## Data Availability

The datasets generated and/or analysed during the current study are not publicly available due personal privacy but are available from the corresponding author on reasonable request.
